# SHR02 Modulates Inflammatory and Oxidative Stress Responses by Inhibiting NF-κB and Activating Nrf2 in Macrophages and Dendritic Cells

**DOI:** 10.4014/jmb.2506.06020

**Published:** 2025-09-23

**Authors:** Hien Thi Thu Do, Chaelin Lee, Inmoo Rhee

**Affiliations:** Department of Bioscience and Biotechnology, Sejong University, Seoul 05006, Republic of Korea

**Keywords:** SHR02, homoisoflavonoid, inflammation, dendritic cells, macrophages, Nrf2

## Abstract

SHR02, a derivative of homoisoflavonoid, exhibits potent anti-inflammatory activity in innate immune cells. In this study, we investigated the immunomodulatory effects of SHR02 in dendritic cells (DC2.4) and macrophages (RAW 264.7) under Toll-like receptor (TLR) stimulation. SHR02 significantly suppressed the secretion of pro-inflammatory cytokines (TNF-α and IL-6), reduced nitric oxide (NO) and reactive oxygen species (ROS) production, and downregulated the expression of iNOS and COX-2. Mechanistically, SHR02 inhibited NF-κB phosphorylation in dendritic cells while enhancing Nrf2 nuclear translocation in both cell types. These findings suggest that SHR02 modulates inflammatory and oxidative responses through both NF-κB and Nrf2 signaling pathways and may serve as a promising candidate for the treatment of inflammatory disorders.

## Introduction

Inflammation is a highly coordinated biological response of the innate immune system to infection, tissue injury, or exposure to harmful stimuli [1, 2]. This process is essential for the elimination of pathogens, the clearance of damaged cells, and the initiation of tissue repair [3-6]. However, when inflammation becomes chronic or dysregulated, it can contribute to the onset and progression of various pathological conditions, including autoimmune diseases, atherosclerosis, neurodegenerative disorders, and cancer [7-9]. The fine balance between protective and pathological inflammation is largely governed by the function of innate immune cells, particularly macrophages and dendritic cells (DCs), which act as sentinels at the front line of host defense [10, 11].

Macrophages and dendritic cells orchestrate inflammatory responses by recognizing pathogen-associated molecular patterns (PAMPs) and damage-associated molecular patterns (DAMPs) through pattern recognition receptors such as Toll-like receptors (TLRs) [12-15]. Among them, TLR4, which recognizes bacterial lipopolysaccharide (LPS), and TLR9, which detects unmethylated CpG DNA, are crucial for initiating robust innate immune responses [16-19]. Activation of TLRs leads to the stimulation of intracellular signaling cascades involving nuclear factor kappa B (NF-κB) and mitogen-activated protein kinases (MAPKs) [20-22]. These pathways culminate in the transcriptional activation of various inflammatory mediators, including pro-inflammatory cytokines (*e.g.*, tumor necrosis factor-α [TNF-α], interleukin-6 [IL-6]), enzymes such as inducible nitric oxide synthase (iNOS) and cyclooxygenase-2 (COX-2), and reactive oxygen/nitrogen species (ROS/RNS) [23, 24]. Sustained or uncontrolled activation of these signaling pathways can result in tissue injury and contribute to inflammatory diseases [1, 25].

In parallel, the nuclear factor erythroid 2–related factor 2 (Nrf2) pathway plays a pivotal role in counterbalancing inflammatory responses by promoting the expression of antioxidant and cytoprotective genes [26, 27]. The crosstalk between NF-κB and Nrf2 signaling has emerged as a crucial regulatory axis in immune homeostasis and inflammation resolution [28-30]. Therapeutic agents that can modulate both pathways represent promising candidates for the treatment of inflammatory diseases.

Natural compounds, including flavonoids and their structural analogs, have gained significant interest in their immunomodulatory, antioxidant, and anti-inflammatory activities. Among these, homoisoflavonoids, a subclass of natural phenolic compounds, have demonstrated potential therapeutic efficacy in various preclinical models. SHR02 is a synthetic derivative of a homoisoflavonoid compound, previously known as SH15002 and reported to possess bioactive properties, yet its immunological effects, particularly on TLR-mediated signaling pathways in innate immune cells, remain poorly understood [31].

In the present study, we sought to investigate the anti-inflammatory and antioxidant effects of SHR02 in macrophages (RAW 264.7) and dendritic cells (DC2.4) upon activation with TLR4 and TLR9 ligands. We evaluated SHR02’s impact on cytokine secretion, nitric oxide (NO) and reactive oxygen species (ROS) production, and the expression of key inflammatory markers such as iNOS, COX-2, NF-κB, and Nrf2. Our findings provide mechanistic insight into the dual modulatory role of SHR02 in innate immune responses and support its potential development as a therapeutic agent for inflammatory and immune-related disorders.

## Materials and Methods

### Cell Culture and SHR02

RAW 264.7 murine cell culture medium includes DMEM (Capricorn, Cat No. DMEM-HPA, Germany), 10%fetal bovine serum (FBS; Corning, Cat No. 35-015-CV, USA) and 1% penicillin-streptomycin (Gibco, Cat No.15140-122, USA). DC2.4 dendritic cells were maintained in RPMI 1640 medium (Capricorn, Cat No. RPMI-A) supplemented with 10% FBS, penicillin-streptomycin, 1% penicillin-streptomycin, L-glutamine. SHR02, formerly known as SH15002, is a homoisoflavonoid compound with antiangiogenic activity, originally derived from botanical sources such as *Chionodoxa luciliae* and *Cremastra appendiculata* (Fig. S1E). Its total synthesis was accomplished through a short synthetic sequence involving 1,4-selective reduction, aldol condensation, and demethylation steps (gifted from Dr. Seung-Yong Seo, Gachon University). This compound was > 95% pure by HPLC analysis.

### Cell Viability Assay

RAW264.7 and DC2.4 cells (4 × 10^5^ cells per well) were incubated for 24 h with or without the drug SHR02 at concentrations of 1, 5, 10, and 20 μM in a 24-well plate. After incubation, cells were harvested, washed with PBS containing 2% FBS, and assessed for viability using the APC Annexin V Apoptosis Detection Kit with 7-AAD (BioLegend, Cat No. 640930, USA). The stained cells were subsequently analyzed using a BD BioScience FACSCantoII cytometer to determine cell viability.

### Nitric Oxide Production Assay

RAW 264.7 or DC2.4 cells (4 × 10^5^ cells per well) were exposed to a concentration gradient of LPS-EK (Invivogen, Cat No. tlrl-eklps, USA) or ODN 1826 (Class B CPG oligonucleotide, Invivogen, Cat No. tlrl-1826) and incubated for 16 h in the presence or absence of SHR02 at concentrations of 1, 5, 10, and 20 μM. Supernatants were collected and analyzed for NO production using the Griess reagent assay. Griess reagent A contained 2% phosphoric acid and 1% sulfanilamide (Sigma-Aldrich, Cat No. S9251, USA); Reagent B contained N-(1-Naphthyl) ethylenediamine dihydrochloride (TCI, Cat No. N0063, Japan). Supernatants were mixed sequentially with reagents A and B and incubated for 10 min in the dark. Absorbance was measured at 540 nm using a SpectraMax M2 microplate reader, and NO concentrations were calculated based on a standard curve.

### Cell Stimulation and Cytokine Detection

RAW 264.7 and DC2.4 cells (5 × 10^4^ cells per well) were stimulated with LPS and SHR02 for 24 h. TNF-α and IL-6 levels in the supernatants were measured using DuoSet^®^ ELISA kits for Mouse TNF-α (Cat No. DY410) and IL-6 (Cat No. DY406) following the manufacturer’s protocols. Reactions were stopped using sulfuric acid (Junsei, Cat No. 83010S0350, Japan), and absorbance was read at 450 nm. All experiments were performed in triplicate.

### Western Blotting

RAW 264.7 and DC2.4 cells (2.5 × 10^6^ per well) were plated in 6-well plates and treated with SHR02 for 20 h. Cells were lysed with cold lysis buffer, and proteins were separated by SDS-PAGE, then transferred to PVDF membranes (GE Healthcare, Cat No. 10600023, USA). After blocking, membranes were incubated with primary antibodies overnight at 4°C or for 1 h at room temperature, followed by HRP-conjugated secondary antibodies (goat anti-mouse or goat anti-rabbit IgG). Protein bands were visualized using enhanced chemiluminescence (Bio-Rad, Cat No. 1705061, USA) and quantified using ImageJ. Protein levels were normalized to β-actin. For cytoplasmic and nuclear fractionation, cells were lysed in TNE buffer and centrifuged to collect the cytoplasmic extract. The pellet was resuspended in RIPA buffer and incubated for 40 min with intermittent vortexing. After centrifugation, the nuclear extract was collected. Both fractions were analyzed by western blot using antibodies against Nrf2, NF-κB, and β-actin. The primary antibodies: β-actin (Santa Cruz, Cat No. sc-47778, 1:500, USA), iNOS (BD Bioscience, Cat No. 610329, 1:500, USA), NF-κB (Santa Cruz, Cat No. sc-8008, 1:200), phosphor-NF-κB (Santa Cruz, Cat No. sc-136548, 1:500), Nrf2 (Cell signaling, Cat No. 12721, 1:1000, USA), corresponding horseradish peroxidase (HRP)-conjugated goat anti-mouse IgG (Jackson ImmunoResearch Laboratories, Cat No. 115-036-003, 1:10000, USA), goat anti-rabbit IgG (Bio-Rad, Cat No. 170-6515, 1:10000).

### Intracellular Reactive Oxygen Species Production Assay

RAW 264.7 and DC2.4 cells (2.5 × 10^6^ cells per well) were seeded in 6-well plates and treated with SHR02 for 20 h. After treatment, cells were washed with DPBS and stained with H2DCFDA (Invitrogen, Cat No. D399, 200 μM) for 30 min. Following a gentle wash, cells were resuspended in DPBS (Welgene, Cat No. 001-02, Korea) and analyzed pusing a BD FACSCanto II flow cytometer (Biopolymer Research Center for Advanced Materials).

### Real-Time Quantitative PCR

Total RNA was isolated using TRIzol reagent (Invitrogen, Cat No. 15596026). cDNA synthesis was performed using the RevertAid First Strand cDNA Synthesis Kit (Thermo Fisher Scientific, Cat No. K1622, USA). qRT-PCR was carried out using PowerSYBR Green PCR Master Mix (Applied Biosystems, Cat No. 4367659) on a StepOne real-time PCR system (Thermo Fisher Scientific). Gene expression levels were calculated using the 2^-ΔCT^ method and normalized to β-actin.

### Statistical Analysis

All experiments were repeated at least three times independently. Data were analyzed using GraphPad Prism 5. Data are presented as mean ± standard deviation (SD). Statistical comparisons among multiple groups were conducted using two-way analysis of variance (ANOVA) to compare each treatment group to the appropriate control.

## Result

### The Inhibitory Function in the Cytokine Production of TNF-α and IL-6 in Innate Immune Cells

To determine the optimal concentration of SHR02 for further experiments, its cytotoxicity was assessed in RAW 264.7 and DC2.4 cells using a cell viability assay. In RAW 264.7 cells, SHR02 treatment at 1-10 μM supported normal cell growth, whereas 20 μM induced cytotoxic effects (Fig. S1). Similarly, in DC2.4 cells, SHR02 at 1–10 μM showed no cytotoxicity, while mild toxicity was observed at 20 μM. Based on these findings, 5–10 μM was selected for investigating the immune regulatory and inhibitory functions of SHR02.

**Fig. 1:**
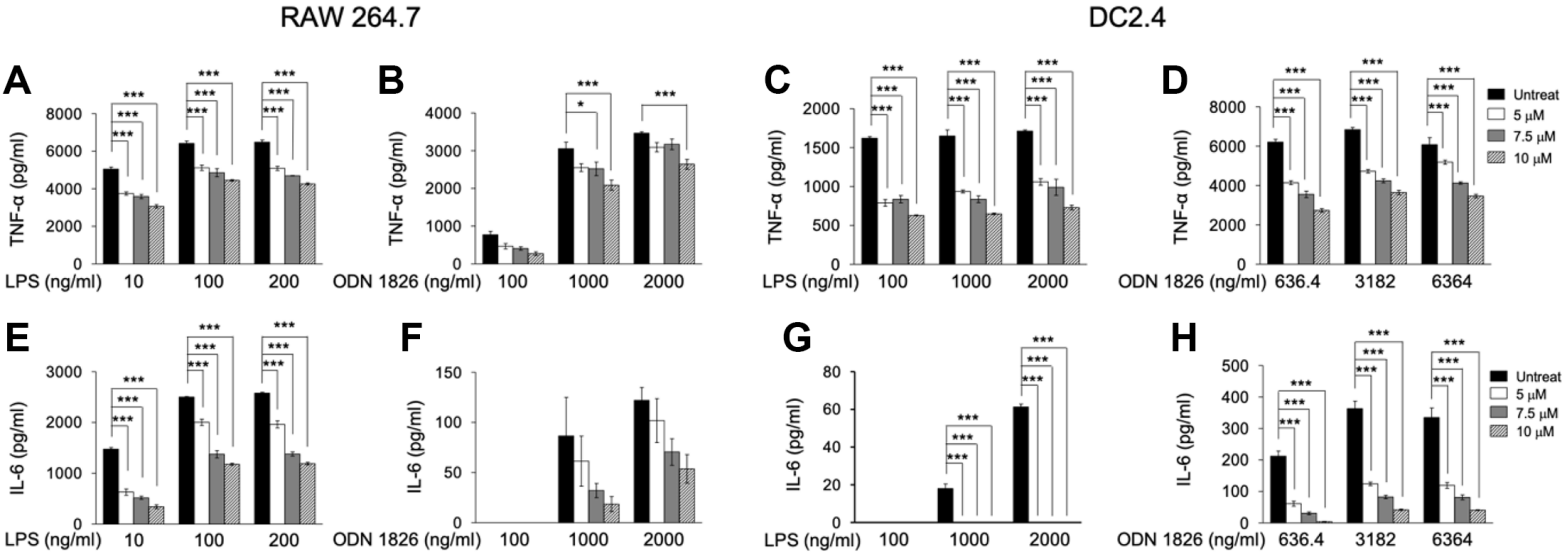
The Effects of SHR02 on TNF-α and IL-6 Production in Macrophages and Dendritic Cells. (**A, B**) Macrophages were stimulated with varying concentrations of TLR agonists (LPS or ODN1826) in the presence of SHR02 (5, 7.5, and 10 μM) for 24 h. (**C, D**) Dendritic cells were stimulated with TLR agonists (LPS or ODN1826) at varying concentrations in the presence of SHR02 (5, 7.5, and 10 μM) for 24 h. TNF-α levels in the supernatant were measured using a TNF-α ELISA kit. (**E, F**) Macrophages were activated with different concentrations of LPS or ODN1826 with or without SHR02 (5, 7.5, and 10 μM) for 24 h. (**G, H**) Dendritic cells were treated with LPS or ODN1826 at different concentrations in the presence or absence of SHR02 (5, 7.5, and 10 μM) for 24 h. In panel G, only the LPS-stimulated group showed a marked increase in IL-6 production, while other groups displayed minimal or undetectable levels, resulting in bars that may appear visually absent. The release of IL-6 in the culture supernatant was measured using an IL-6 ELISA kit. Data are presented as mean ± SD from triplicate cultures, with statistical significance determined by ANOVA test (*p < 0.05, **p < 0.01, ***p < 0.001 vs. SHR02-untreated group).

During inflammation, macrophages are key effector cells recruited to injury sites, releasing inflammatory mediators that drive the inflammatory response [32]. To evaluate the effects of SHR02 on macrophage-mediated inflammation, the secretion of TNF-α and IL-6 was measured in RAW 264.7 cells activated by TLR agonists (LPS, ODN 1826) and treated with SHR02 at the concentration 5, 7.5, or 10 μM for 24 h. ELISA results showed that SHR02 had a limited capacity to inhibit TNF-α and IL-6 secretion in TLR-engaged macrophages (Fig. 1). At 100 ng/ml LPS and 3,182 ng/ml ODN 1826, macrophages produced high levels of TNF-α (Fig. 1A and 1B). SHR02 attenuated TNF-α production in a dose-dependent manner, with the highest inhibition (~20-30%) observed at 10 μM in both LPS- and ODN 1826-activated cells. Similarly, IL-6 secretion was significantly reduced, with 10 μM SHR02 decreasing IL-6 levels by over 50% compared to untreated cells (Fig. 1E and 1F.).

Dendritic cells also contribute to inflammation by producing pro-inflammatory cytokines upon TLR activation [33]. To determine whether SHR02 modulates cytokine secretion in DCs, DC2.4 cells were stimulated with LPS or ODN 1826 in the presence of SHR02 (5, 7.5, or 10 μM). SHR02 effectively suppressed TNF-α and IL-6 secretion in a concentration-dependent manner (Fig. 1). In LPS-activated DCs, TNF-α production was reduced by more than half at 10 μM (Fig. 1C), while inhibition was slightly lower in ODN 1826-stimulated cells (Fig. 1D). SHR02 also demonstrated a strong inhibitory effect on IL-6 secretion in LPS-stimulated DC2.4 cells (Fig. 1G). Notably, in TLR9-activated DCs, IL-6 production was reduced by more than 90% at 10 μM SHR02 (Fig. 1H). These findings indicate that SHR02 effectively modulates inflammation by suppressing pro-inflammatory cytokine secretion in both macrophages and dendritic cells.

### The Inhibitory Ability of SHR02 to Nitric Oxide Production in Macrophages and Dendritic Cells

**Fig. 2:**
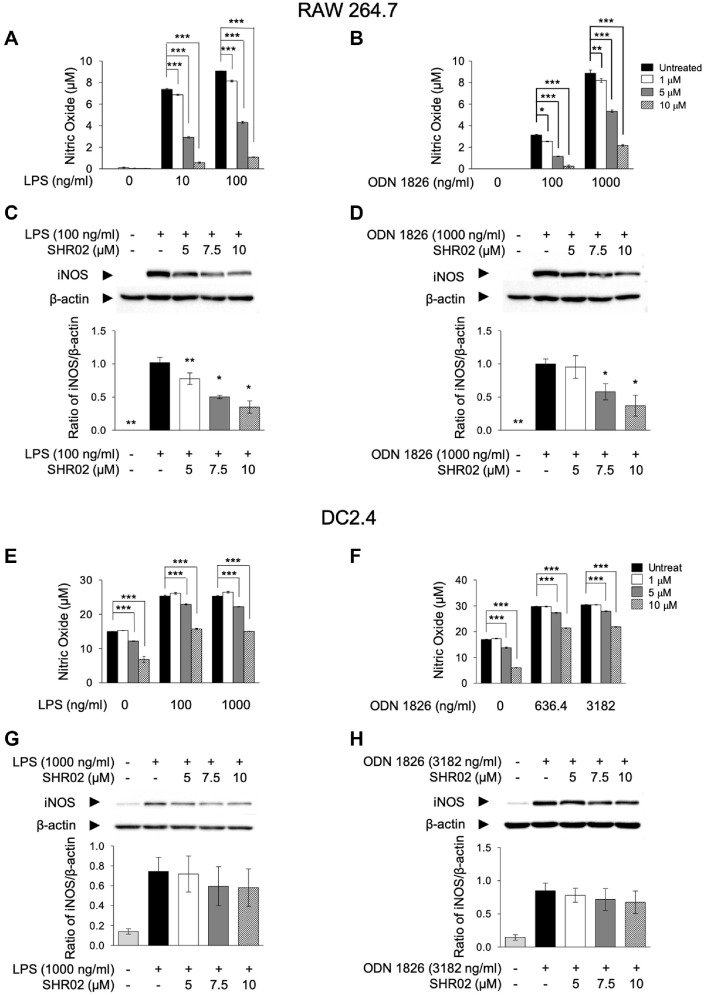
The modulatory ability of SHR02 on Nitric Oxide (NO) Production in innate immune cells. (**A, B**) Macrophages were treated with LPS (100 ng/ml) or ODN 1826 (1,000 ng/ml), and SHR02 at increasing concentrations (5, 7.5, and 10 μM) for 16 h. (**E, F**) Dendritic cells were stimulated with LPS (1,000 ng/ml), ODN 1826 (3,182 ng/ml) in the presence of SHR02 (5, 7.5, and 10 μM) for 16 h. The nitric oxide (NO) production was assessed in the cell culture supernatant. Data are shown as mean ± SEM from triplicate supernatant measurements. Statistical significance was determined using two-way ANOVA (*p < 0.05, **p < 0.01, ***p < 0.001 vs. SHR02-untreated group). (**C, D**) Macrophages were treated with LPS (100 ng/ ml), ODN 1826 (1,000 ng/ml) in combination with SHR02 at different concentrations for 20 h. (**G, H**) Dendritic cells were treated with LPS (1,000 ng/ml), ODN 1826 (3,182 ng/ml) in the presence of SHR02 at different concentrations for 20 h. Cells were lysed, and total protein extracts were subjected to western blot analysis using an anti-iNOS antibody. The blots were quantified using ImageJ, with iNOS expression levels normalized to β-actin. Representative blot images are displayed. Statistical significance was determined using an ANOVA test (*p < 0.05 vs. SHR02-untreated group).

Macrophages produce various inflammatory mediators and regulatory enzymes, including nitric oxide and inducible nitric oxide synthase [34]. To evaluate the inhibitory effects of SHR02 on NO production and iNOS expression, RAW 264.7 cells were activated with TLR agonists (LPS or ODN 1826) and treated with SHR02 at different concentrations. LPS (100 ng/ml) and ODN 1826 (1,000 ng/ml) significantly increased NO production in RAW 264.7 cells (Fig. 2A and 2B). However, SHR02 (10 μM) exhibited potent inhibitory activity, reducing NO levels by over 80% compared to untreated cells. To investigate the NO signaling pathway, iNOS expression was analyzed in RAW 264.7 cells treated with LPS (100 ng/ml) or ODN 1826 (1,000 ng/ml) alongside SHR02. Similarly, iNOS expression was upregulated in response to TLR stimulation (Fig. 2C and 2D), while SHR02 suppressed iNOS expression in a dose-dependent manner.

Since NO is also a key inflammatory marker in dendritic cells under infectious conditions [35], the effects of SHR02 on NO production were further investigated in DC2.4 cells. TLR stimulation with LPS or ODN 1826 increased NO production, whereas SHR02 significantly reduced NO release in a concentration-dependent manner (Fig. 2E and 2F). In western blot results detecting iNOS levels, both TLR4 and TLR9 activation induced iNOS expression, but SHR02-treated groups exhibited a moderate reduction in iNOS levels, depending on the concentration (Fig. 2G and 2H).

These findings suggest that SHR02 effectively attenuates inflammation by inhibiting NO production and suppressing iNOS expression in macrophages and dendritic cells.

### The Effect of the Drug SHR02 on the COX-2 Production in Innate Immune Cells

**Fig. 3:**
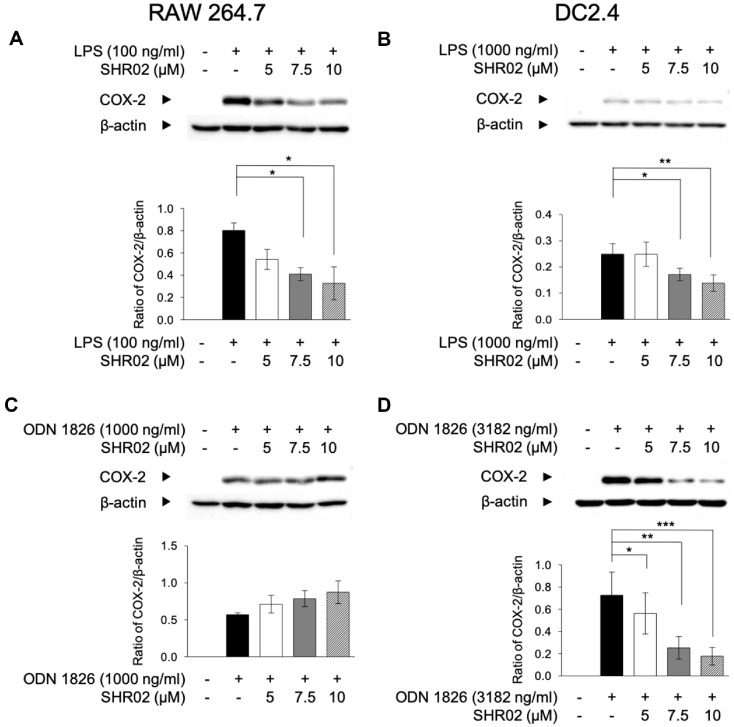
The effect of SHR02 on COX-2 expression in macrophages and dendritic cells. (**A, B**) Macrophages were activated with LPS (100 ng/ml) or with ODN 1826 (1,000 ng/ml) in the presence of SHR02 at increasing concentrations (5, 7.5, and 10 μM) for 20 h. (**C, D**) Dendritic cells were treated with LPS (1,000 ng/ml) or ODN 1826 (3,182 ng/ml), and SHR02 (5, 7.5, and 10 μM) for 20 h. The expression of COX-2 was determined by western blotting using an anti-COX-2 antibody. ImageJ was used to quantify the blots, and COX-2 expression was normalized to β-actin. Representative blot images are shown, and results are presented as mean ± SD from three independent experiments. Statistical significance was analyzed using an ANOVA test (*p < 0.05, **p < 0.01 vs. SHR02-untreated group).

In the inflammatory process, cyclooxygenase-2 plays a crucial role in macrophages by regulating downstream inflammatory mediators [36]. To determine whether SHR02 affects COX-2 expression under TLR stimulation, RAW 264.7 cells were treated with varying concentrations of SHR02 following stimulation with LPS or ODN 1826 and analyzed for COX-2 protein levels. Both LPS (100 ng/ml) and ODN 1826 (1,000 ng/ml) significantly upregulated COX-2 expression (Fig. 3A and 3C). However, SHR02 effectively suppressed COX-2 expression in LPS-activated macrophages in a dose-dependent manner with 7.5 μM reducing expression by more than 50%. In contrast, SHR02 had no significant effect on COX-2 expression in ODN 1826-stimulated macrophages (Fig. 3C).

To further evaluate the anti-inflammatory properties of SHR02, its effects on COX-2 expression were also examined in DC2.4 cells. COX-2 expression was significantly upregulated in TLR-agonist-treated groups (Fig. 2B and 2D), while SHR02 reduced its expression in a dose-dependent manner. Notably, at 10 μM, SHR02 decreased COX-2 levels by over 70% in ODN 1826-treated DC2.4 cells (*P* < 0.01, Fig. 3D). These findings suggest that SHR02 effectively attenuates inflammation by modulating COX-2 expression in macrophages and dendritic cells, particularly under TLR4 activation.

### The Influence of SHR02 on the ROS Induction in Macrophages and Dendritic Cells

**Fig. 4:**
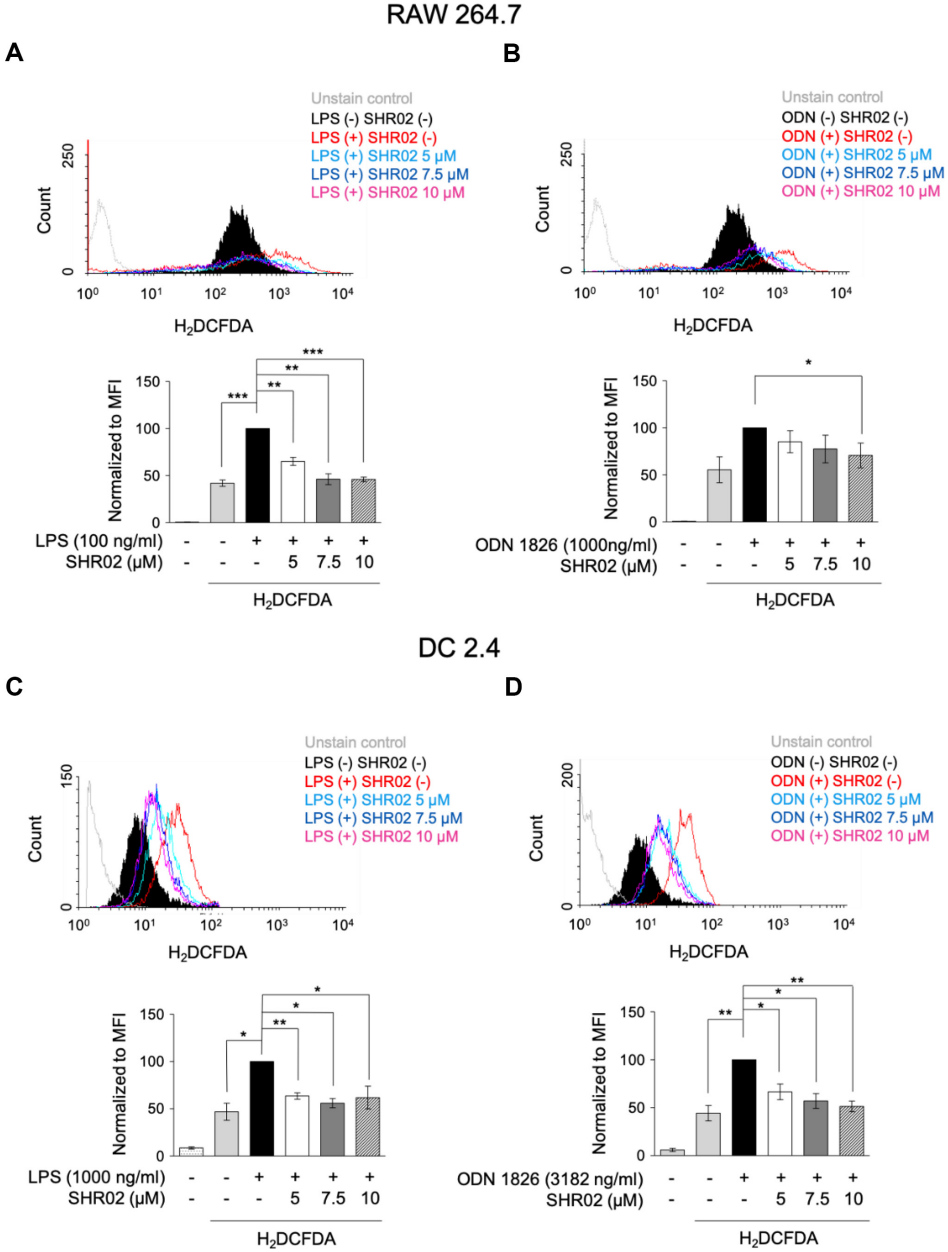
The inhibitory ability of SHR02 on ROS production in macrophages and dendritic cells. (**A, B**) Macrophages were activated with LPS (100 ng/ml) or ODN 1826 (1,000 ng/ml). (**C, D**) Dendritic cells were activated with LPS (1,000 ng/ml) or ODN 1826 (3,182 ng/ml). Both immune cells were co-stimulated in the presence of SHR02 at 5, 7.5, and 10 μM for 20 h. ROS generation was assessed using H2DCFDA staining, and fluorescence intensity was measured by flow cytometry. Representative histograms are displayed, and the mean fluorescence intensity (MFI) is presented as mean ± SD from three independent experiments. Statistical differences were analyzed using an ANOVA test (*p < 0.05, **p < 0.01 vs. SHR02- untreated group).

ROS plays a crucial role in inflammatory signaling, as TLR agonists like LPS and CpG (ODN 1826) enhance ROS production, leading to oxidative stress and inflammation [37]. To investigate the immunomodulatory effects of SHR02, RAW 264.7 cells were treated with LPS or ODN 1826 in the presence of SHR02. After 20 h, cells were harvested, and reactive oxygen species production was assessed using H2DCFDA fluorescence. In TLR4-stimulated cells (Fig. 4A), ROS generation was significantly elevated compared to the control group. However, treatment with SHR02 at 7.5–10 μM effectively reduced ROS levels to baseline. In contrast, SHR02 had no significant effect on ROS production in ODN 1826-stimulated RAW 264.7 cells (Fig. 4B).

To determine whether SHR02 can suppress ROS accumulation dendritic cells, DC2.4 cells were treated with LPS (1,000 ng/ml) or ODN 1826 (3,182 ng/ml) in the presence or absence of SHR02 (5, 7.5, and 10 μM). LPS-stimulated DC2.4 cells exhibited a significant increase in ROS production, whereas SHR02 treatment led to a slight, concentration-dependent reduction in ROS levels (Fig. 4C). Similarly, ODN 1826 stimulation resulted in elevated ROS levels, but SHR02 effectively suppressed ROS generation (Fig. 4D). These findings suggest that SHR02 mitigates inflammation by reducing ROS production, particularly in TLR4- and TLR9-stimulated DCs.

### The Influence of SHR02 on the NF-κB-Related Signaling Pathway in Innate Immune Cells

**Fig. 5:**
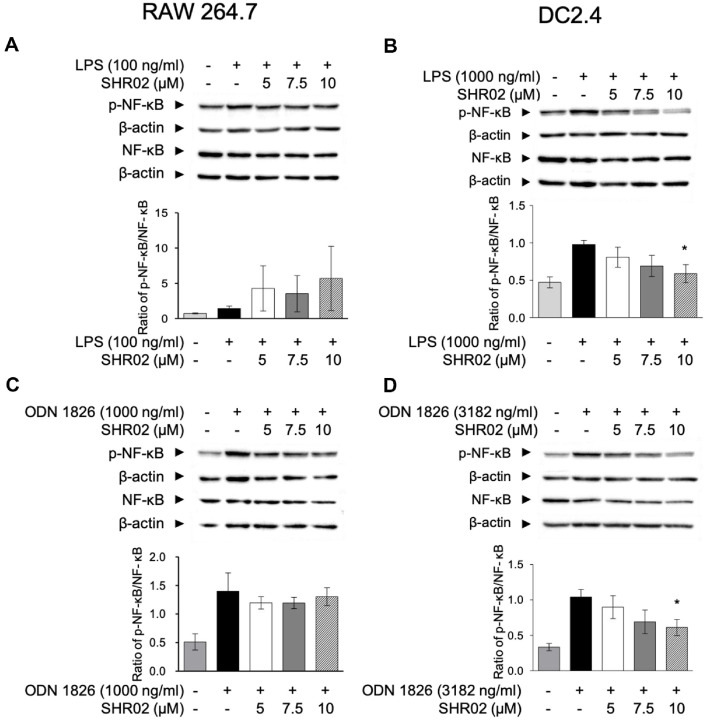
The effect of SHR02 on the phosphorylation of NF-κB in macrophages and dendritic cells. (**A, B**) RAW 264.7 cells were activated with LPS (100 ng/ml) or ODN 1826 (1,000 ng/ml). (**C, D**) Dendritic cells were activated with LPS (1,000 ng/ml) or ODN 1826 (3,182 ng/ml). Both innated immune cells were co-treated with SHR02 at increasing concentrations. After 20 h, cell lysates were prepared using RIPA buffer and analyzed by western blot with anti-NF-κB and anti-phospho-NF- κB antibodies. β-actin was used as a loading control. Blots were quantified using ImageJ, and data are presented as mean ± SD from three independent experiments. Statistical analysis was performed using an ANOVA test (*p < 0.05 vs. unstimulated control). Representative blot images are displayed.

Both TLR4 and TLR9 stimulation activate the NF-κB signaling pathway, playing a crucial role in inflammation, initiating the expression of pro-inflammatory mediators in various immune cells, including macrophages and DCs [38]. To determine whether SHR02 influences this pathway, RAW 264.7 cell lysates were analyzed for NF-κB phosphorylation after 20 h of stimulation with TLR agonists and SHR02. The western blotting result indicated SHR02 did not significantly enhance or suppress NF-κB phosphorylation in either LPS- or ODN 1826-activated groups (Fig. 5A and 5C).

Similarly, TLR agonists activate NF-κB to induce cytokine release, inhibiting NF-κB signaling is a potential strategy for regulating inflammation in DCs [39]. Therefore, the impact of SHR02 on NF-κB phosphorylation was further assessed in DC2.4 cells. LPS and ODN 1826 stimulation led to increased NF-κB phosphorylation (Fig. 5B and 5D). However, SHR02 treatment reduced phospho-NF-κB expression in a dose-dependent manner. At 10 μM, SHR02 significantly suppressed NF-κB phosphorylation by more than 40% (Fig. 5D).

These findings suggest that while SHR02 does not affect NF-κB phosphorylation in RAW 264.7 macrophages, it effectively downregulates NF-κB activation in DC2.4 cells, indicating a potential role in modulating dendritic cell-mediated inflammation through NF- κB signaling pathway.

### The Influence of SHR02 on the Nrf2 Expression in Macrophages and Dendritic Cells

**Fig. 6:**
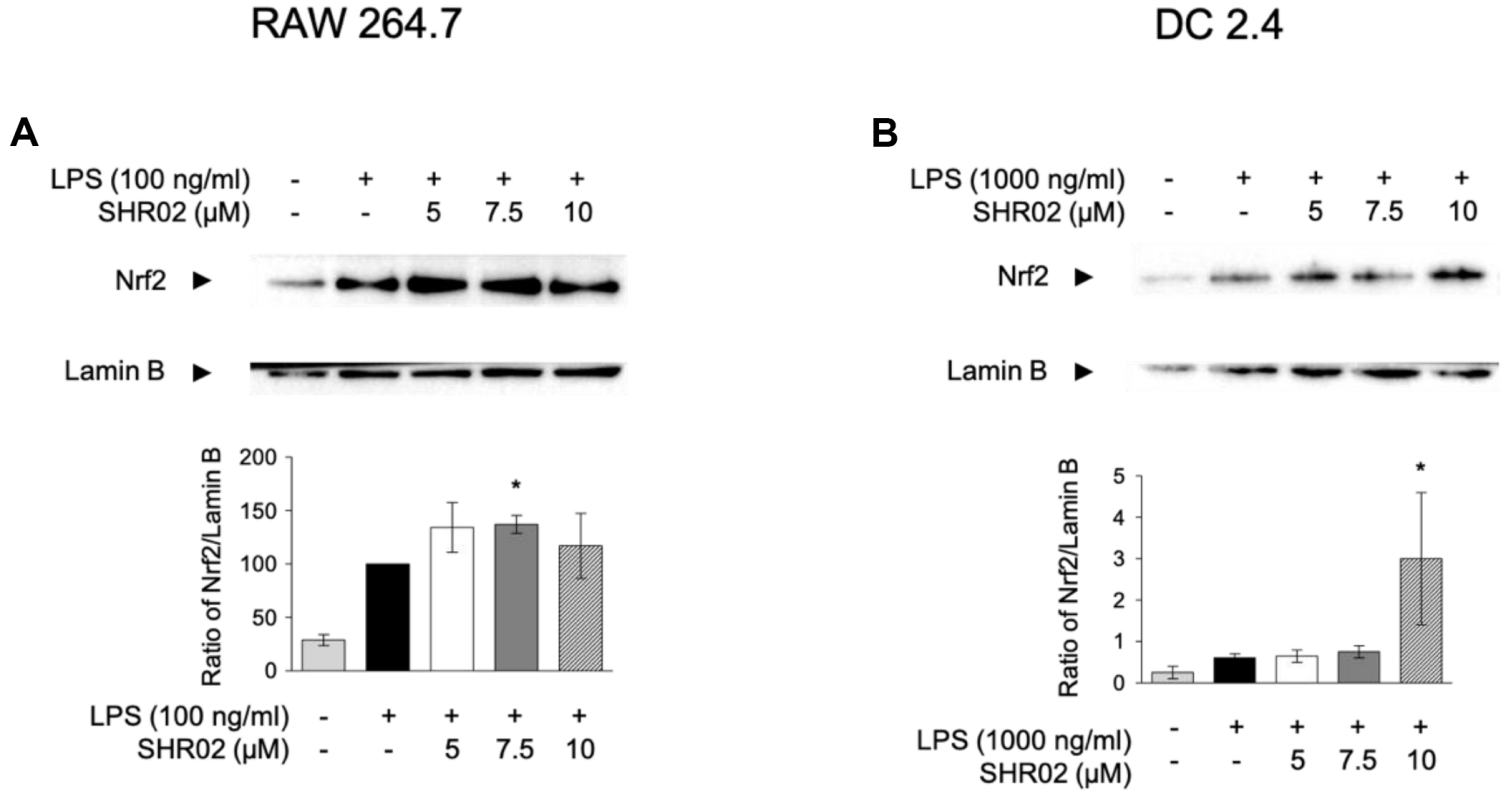
The stimulating activity of SHR02 on nuclear translocation of Nrf2 in macrophages and dendritic cells. (**A**) RAW 264.7 cells were treated with SHR02 in a dose-dependent manner alongside LPS (100 ng/ml) for 20 h. (B) DC2.4 cells were treated with SHR02 in a dose-dependent manner alongside LPS (1,000 ng/ml) for 150 min. Following incubation, nuclear and cytoplasmic fractions were extracted and analyzed via western blot using an anti-Nrf2 antibody. β- actin was used as a loading control. Representative blot images are shown. Nrf2 expression levels were quantified using ImageJ, and data are presented as mean ± SD from three independent experiments. Statistical differences were analyzed using an ANOVA test (*p < 0.05, **p < 0.01 vs. unstimulated control).

Nrf2 is a key transcription factor that regulates inflammatory responses in immune cells [27]. The activation of the Nrf2 signaling pathway upregulates the transcription of antioxidant genes, leading to the modulation of inflammatory mediators [29]. To investigate the intracellular mechanism of SHR02, its effect on Nrf2 expression in macrophages was evaluated. RAW 264.7 cells were co-treated with LPS (100 ng/ml) and varying concentrations of SHR02 (5, 7.5, and 10 μM) for 20 h. After treatment, nuclear extracts were collected for western blot analysis. LPS stimulation alone increased Nrf2 expression, while SHR02 further enhanced Nrf2 levels ccompared to the LPS-only group (Fig. 6A). Notably, SHR02 at 5, 7.5 μM increased Nrf2 expression by more than 50% compared to baseline levels.

To determine whether SHR02 also influences Nrf2 signaling in dendritic cells, DC2.4 cells were treated with TLR agonists and SHR02 for 150 min, followed by nuclear extraction and western blot analysis. The result demonstrated that LPS alone upregulated Nrf2 expression, and SHR02 at 10 μM further enhanced nuclear Nrf2 levels (Fig. 6B).

These findings suggest that SHR02 enhances Nrf2 expression in both macrophages and dendritic cells, potentially contributing to its immune-modulatory effects through the regulation of antioxidant and inflammatory responses.

## Discussion

This study comprehensively demonstrates that SHR02, a homoisoflavonoid derivative, exhibits broad anti-inflammatory and antioxidant activities in two major innate immune cell types: macrophages and dendritic cells. By employing TLR4 and TLR9 stimulation models, we observed that SHR02 significantly inhibited the production of key inflammatory mediators including TNF-α, IL-6, nitric oxide (NO), and ROS in both RAW 264.7 and DC2.4 cells.

Notably, SHR02 reduced cytokine secretion in a dose-dependent manner, with stronger inhibition of IL-6 than TNF-α, particularly in TLR9-activated dendritic cells. The suppression of iNOS and COX-2 expression provides further evidence that SHR02 effectively downregulates inflammatory enzyme expression at the protein level. These results indicate that SHR02 not only interferes with cytokine release but also blocks key biosynthetic enzymes involved in inflammatory cascades.

Mechanistically, SHR02 was found to exert distinct effects on intracellular signaling pathways. While NF-κB phosphorylation was unaffected in RAW 264.7 macrophages, it was significantly suppressed in DC2.4 cells, suggesting a cell-type-specific regulatory mechanism. This differential response may reflect intrinsic differences in TLR signaling dynamics and sensitivity between macrophages and dendritic cells, as well as variations in upstream regulators or feedback mechanisms influencing NF-κB activation.

In addition to its anti-inflammatory role, SHR02 activated the Nrf2 pathway in both macrophages and dendritic cells, as demonstrated by enhanced nuclear translocation of Nrf2. Nrf2 is a master regulator of antioxidant gene expression and cellular redox balance [40], and its activation further supports the cytoprotective and homeostasis-restoring role of SHR02 in inflammatory conditions.

The dual action of SHR02-suppressing NF-κB-driven inflammation while enhancing Nrf2-mediated antioxidant responses positions it as a promising candidate for managing chronic inflammatory diseases, especially those involving innate immune dysregulation such as sepsis, inflammatory bowel disease, or autoimmune conditions. Importantly, the activity profile of SHR02 across both macrophages and dendritic cells suggests that it could dampen innate immune overactivation at multiple checkpoints. Compared to other natural product derivatives, SHR02 demonstrates a favorable balance of immunomodulation without overt cytotoxicity at effective concentrations. The concentration range of 1-10 μM used in this study was based on cell viability data and prior studies involving structurally related homoisoflavonoid compounds. These doses fall within a range that is pharmacologically achievable in vitro and allow for the assessment of the immunomodulatory effects of SHR02 without inducing cytotoxicity. Future in vivo studies and pharmacokinetic profiling are warranted to evaluate its clinical translational potential. Nonetheless, a limitation of this study is the absence of in vivo validation, which is essential for determining the therapeutic efficacy of SHR02, pharmacokinetics, and potential toxicity in a physiological context. Future animal studies will be critical to establish its translational potential and confirm its immunomodulatory effects in vivo. SHR02 effectively regulates inflammatory signaling in innate immune cells by inhibiting TLR-induced cytokine release, nitric oxide and ROS production, and by modulating NF-κB and Nrf2 pathways. Its selective inhibition in dendritic cells and consistent antioxidant-enhancing effects make it a promising candidate for anti-inflammatory drug development.
